# Synthesis of Radiation Curable Palm Oil–Based Epoxy Acrylate: NMR and FTIR Spectroscopic Investigations

**DOI:** 10.3390/molecules200814191

**Published:** 2015-08-04

**Authors:** Ashraf M. Salih, Mansor Bin Ahmad, Nor Azowa Ibrahim, Khairul Zaman Hj Mohd Dahlan, Rida Tajau, Mohd Hilmi Mahmood, Wan Md. Zin Wan Yunus

**Affiliations:** 1 Department of Chemistry, Faculty of Science, University Putra Malaysia, UPM, Serdang 43400, Selangor, Malaysia; E-Mail: norazowa@science.upm.edu.my; 2 Department of Radiation Processing, Sudan Atomic Energy Commission, Khartoum 1111, Sudan; 3 Polycomposite Sdn Bhd, No.75-2, Jalan TKS 1, Taman Kajang Sentral, Kajang 43000, Selangor, Malaysia; E-Mail: dr.khairulz@gmail.com; 4 Radiation Processing Technology Division, Nuclear Malaysia, Bangi, Kajang 43000, Selangor, Malaysia; E-Mail: rida@nuclearmalaysia.gov.my; 5 No.107, Jalan 2, Taman Kajang Baru, Sg Jelok, Kajang 43000, Selangor, Malaysia; E-Mail: hilmi_mahmood@yahoo.com; 6 Department of Chemistry, Centre for Defence Foundation Studies, National Defence University of Malaysia, Sungai Besi Camp 57000, Kuala Lumpur, Malaysia; E-Mail: wanmdzin@upnm.edu.my

**Keywords:** palm oil, epoxy acrylate, UV-curing, NMR, FTIR

## Abstract

Over the past few decades, there has been an increasing demand for bio-based polymers and resins in industrial applications, due to their potential lower cost and environmental impact compared with petroleum-based counterparts. The present research concerns the synthesis of epoxidized palm oil acrylate (EPOLA) from an epoxidized palm oil product (EPOP) as environmentally friendly material. EPOP was acrylated by acrylic acid via a ring opening reaction. The kinetics of the acrylation reaction were monitored throughout the reaction course and the acid value of the reaction mixture reached 10 mg KOH/g after 16 h, indicating the consumption of the acrylic acid. The obtained epoxy acrylate was investigated intensively by means of FTIR and NMR spectroscopy, and the results revealed that the ring opening reaction was completed successfully with an acrylation yield about 82%. The UV free radical polymerization of EPOLA was carried out using two types of photoinitiators. The radiation curing behavior was determined by following the conversion of the acrylate groups. The cross-linking density and the hardness of the cured EPOLA films were measured to evaluate the effect of the photoinitiator on the solid film characteristics, besides, the thermal and mechanical properties were also evaluated.

## 1. Introduction

Bio-based polymers and resins are currently attracting a great deal of interest in both research and industrial applications. Such polymers have many advantages compared to conventional petroleum-based polymers. They are biodegradable, and can be obtained from renewable resources at lower cost. Vegetable oils are composed of complex mixtures of different triglycerides, which are esters of glycerol and fatty acids [1]. Triglycerides are one of the most important raw materials for bio-based thermosetting polymers [2]. One of the most effective modifications of triglycerides is the epoxidation reaction, in which the C=C double bonds are converted to oxirane (epoxide) rings [3] which are highly strained and very reactive. They can easily undergo variety of reactions to give important chemical compounds for polymer synthesis, such as polyols [4], and can undergo cationic polymerization to form polymeric networks [5], as well as be used in UV curable systems [6].

Epoxidized oil acrylate is synthesized by several methods; Habib and Bajpai reported the synthesis of epoxidized soybean acrylate using acrylic acid in the acrylation reaction. Analytical and spectroscopic characterization of the product revealed that the epoxy rings were opened and the epoxy acrylate was obtained successfully [7]. Pelletier and Gandini synthesized epoxy acrylates by reacting the hydroxyl and oxirane functional groups in the triglyceride molecules with acryloyl chloride [8]. Castor oil was found to be a good renewable source for polymeric material [9]. Vernonia oil is a naturally epoxidized oil. It was used to prepare epoxy acrylates by reacting the oxirane rings with acrylic and methacrylic acids and acrylation yields were up to 85%–98% [10].

Compared to conventional curing methods, photopolymerization (UV) curing could be an excellent alternative method to initiate the polymerization reaction in monomer systems, or to induce cross-linking polymerization in the polymer matrix. Drawbacks of the conventional methods are the need for toxic chemicals for chemical curing, or the high thermal energy used in thermal curing. The slow curing rates could be avoided by the UV curing method, which therefore, it is an environmentally friendly process that could be performed at low temperature, with a faster curing rate [11]. Over the past decades, the UV radiation curing technique has become a technique used worldwide, with a minimum usage of toxic volatile materials [12]. UV curing systems basically consist of oligomers, photoinitiators and low pressure monomers, which are used as reactive diluents to adjust the viscosity of the curing systems [12,13], as well as to introduce certain properties to the cured films, for instance, improving the chemical resistance, hardness, flexibility, durability and adhesion to the substrates [14]. The UV radiation curing technique has been applied in different curable acrylate system applications, such as biomedical applications and dental uses [15], polymer electrolytes for lithium batteries [16,17], polymer films for electronic circuit protection [18], ion-exchange membranes [19], and extensively in coating applications and adhesives [20,21,22], and showed excellent results with high performance cured materials.

Epoxidized palm oil was successfully acrylated by acrylic acid on a pilot scale and the results were compared with the laboratory scale results. It was reported that both products have a potential to be applied as a radiation curable systems [23].

Several pervious articles have reported the synthesis of epoxidized palm oil acrylate for different purposes and applications [24,25]. However, the chemical reactions during the synthesis process, and the chemical structure of the final product, need more investigation with a powerful analytical technique such as the NMR, to provide more detailed information. Thus, this article aims to present the synthesis of epoxidized palm oil acrylate in detail, using an epoxidized palm oil product (EPOP) as raw material, and provides a comprehensive spectroscopic investigation of the ring opening (acrylation) reaction, in addition, to study the UV radiation curing behavior of this resin, using two different types of photoinitiators.

## 2. Results and Discussion

### 2.1. Epoxidized Palm Oil Acrylate (EPOLA)

**Scheme 1:**
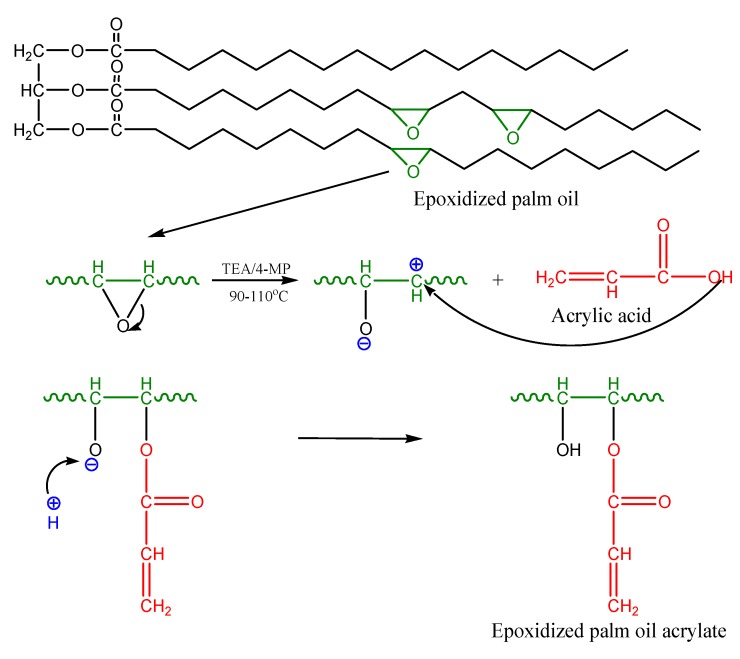
Mechanism of the acrylation reaction of epoxidized palm oil by acrylic acid.

EPOLA was successfully synthesized by an esterification reaction between epoxidized palm oil product (EPOP) and acrylic acid in the presence of triethylamine (TEA) as a catalyst. The epoxy groups (oxirane rings) reacted with the carboxylic acid groups in the acrylic acid to form a hydroxy acrylate resin. Scheme 1 shows a diagram of the proposed reaction mechanism. 

**Figure 1:**
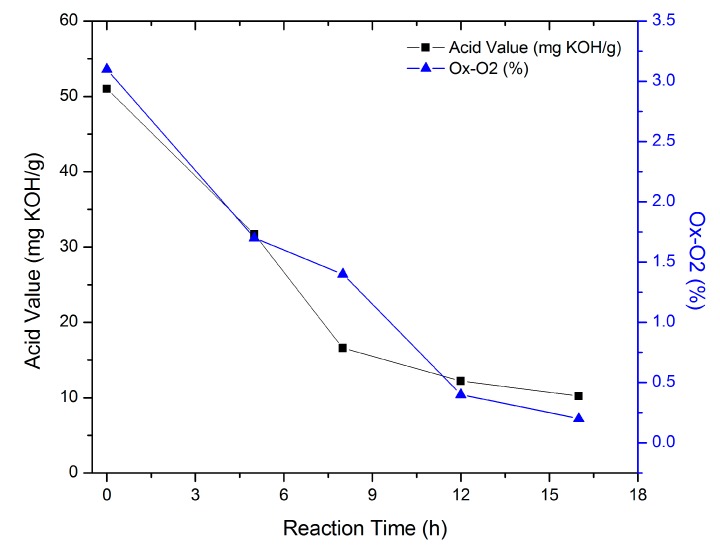
EPOLA Synthesis, Acid values and O_x_-O_2_ (%) *vs* Acrylation Reaction Time.

The acrylation reaction involves the consumption of the carboxylic groups of the acrylic acid and the epoxy rings; therefore, the reaction progress was followed by measuring the acid value of the reaction matrix. Figure 1 shows the decrease of the acid values and the oxirane ring percentages during the reaction course. The initial acid value was 51 mg KOH/g, which started to decrease rapidly in the first 8 h, followed by a slow decrease in the successive hours, and this could be attributed to the elevated concentrations of acrylic acid and oxirane groups during the first hours, and thus higher reactivity, which decreased as the reaction progressed. The reaction was stopped when the acid value was about 10.2 mg KOH/g. The FTIR and NMR results revealed the consumption of the acrylic acid, as well as the formation of new acrylate functional groups. 

The physical and analytical characteristics of EPOP and EPOLA are presented in Table 1. The decrease of the percentage of oxirane rings reveals the consumption of epoxy groups as a result of the acrylation process. On the other hand, the addition of the acrylate groups to the epoxy chain resulted in a higher molecular weight product (EPOLA), with a higher viscosity compared to the starting reactant EPOP. EPOLA was purified by washing with distilled water, whereby a lower acid value product (about 1.5 mg KOH/g) was obtained, which is due to the elimination of the unreacted acrylic acid.

**Table 1:** Physio-chemical characteristics of EPOP and EPA.

Resin	Acid Value (mg KOH/g)	Iodine Value (I_2_/100g)	OH-Value (mg KOH/g)	Ox-O_2_%/100 g	Viscosity (cPs) at 25 °C	Density (g/cm^3^)
EPOP	2.5	0.76	12.9	3.0	280	0.9095
EPOLA	10.2	20.7	50.99	0.2	580	0.9748
Purified EPOLA	1.5	18.6	44.9	---	1350	0.9941

The molecular weight and molecular weight distribution of EPOP and EPOLA were determined by GPC, and the polydispersity index (PDI) was calculated from the ratio of the weight average molecular weight$\;(\bar{M_{w}}$) to the number average molecular weight $\left(\bar{M_{n}}\right)$, using Equation (1):

(1) $$PDI=\;\frac{\bar{M_{w}}}{\bar{M_{n}}}$$

**Figure 2:**
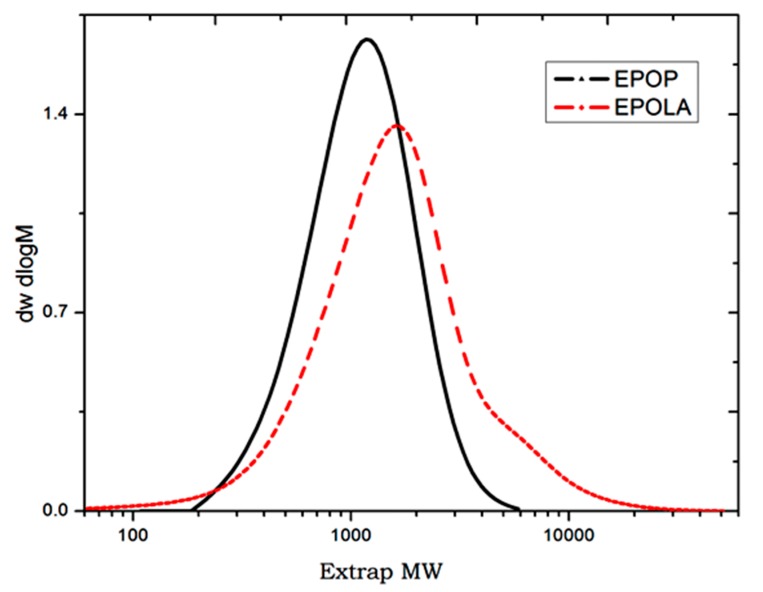
Molecular Weight Distribution Curves of EPOP and EPOLA.

Figure 2 shows the molecular weight distribution of EPOP and EPOLA. It could be observed that both EPOP and EPOLA have monomodal distribution curves, which indicates the homogeneity of the length of the resin molecules. On the other hand, the molecular weight of EPOP was increased due to the extent of the chains increasing by grafting of acrylate groups onto the triglyceride backbone. These results are in good agreement with the increase of the viscosity, as it could be seen in Table 1 and Table 2.

**Table 2:** Molecular weights, PDI of EPOP and EPOLA.

Resin	M_w_ (g/mol)	M_n_ (g/mol)	PDI (M_w_/M_n_)
EPOP	1262	925	1.3
EPOLA	1667	1053	1.5

**Figure 3:**
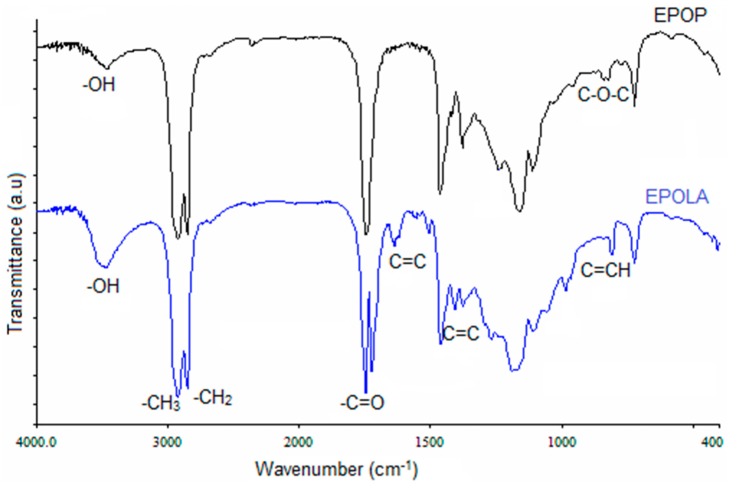
FTIR Spectra of EPOP and EPOLA.

The functional groups of EPOP and EPOLA were determined by FTIR spectroscopy and results are shown in Figure 3. The FTIR spectra of EPOP shows clearly the stretching vibration of C-O-C groups at 1240 cm^−1^, in addition to the other characteristic band of the epoxy rings at the wavenumber between 850–830 cm^−1^. The terminal methyl groups (CH_3_) of the triglyceride chains showed a strong band of C-H stretching at 2924 cm^−1^, while the methylene moieties (CH_2_) in the saturated fatty acid backbone show a stretching band at 2853 cm^−1^, and a C-H in-plane deformation band at 1378 cm^−1^.

Obviously, the comparison between the FTIR spectra of EPOP and EPOLA reveals that there are new IR absorption bands in the spectrum of EPOLA, such as the absorption band at 810 cm^−1^, which is attributed to the out-of-plane deformation of the C=C of the vinyl moieties of the acrylate groups, besides, the band at 1636 cm^−1^. Table 3 shows the FTIR assignments of the major functional groups of EPOP and EPOLA.

**Table 3:** FTIR peaks assignment of EPOP and EPOLA.

Functional Group	FTIR Absorption (cm^−1^)	
	EPOP	EPOLA
OH stretching	3472	3470
C-H stretching (CH_3_)	2924	2924
C-H stretching (CH_2_)	2853	2853
C=O stretching	1743	1744
C=O (carboxylic group)	-----	1725
CH_2_=CH-R stretching	-----	1636
CH_2_=CH-R Scissoring	-----	1406
C=O stretching	1743	1744
C=O (carboxylic group)	-----	1725
CH_2_=CH-R stretching	-----	1636
CH_2_=CH-R Scissoring	-----	1406
C-O-C stretching	1240	-----
C-O-C asymmetric bending	835	-----
CH=C-H out of plane bending	------	810

### 2.2. NMR Analysis of EPOP and EPOLA

**Figure 4:**
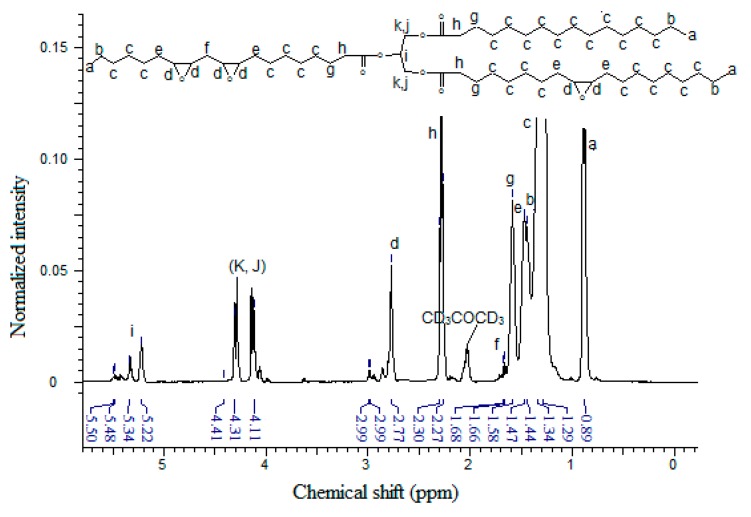
^1^H-NMR Spectrum of EPOP.

The structures of EPOP and EPOLA were investigated by NMR spectrometry. For this the resins were dissolved in deuterated acetone (acetone-d_6_) and both ^1^H-NMR and ^13^C-NMR spectra were recorded. Figure 4 shows the ^1^H-NMR spectrum of EPOP, where the acetone-d_6_ signal appears at (δ) 2.00 ppm. The characteristic peaks of the triglyceride molecule can be observed clearly; for instance, the triplet peak signal with an integration corresponding to nine protons at (δ) 0.89 ppm of the terminal methyl protons –C**H**_3_ (Ha), and the protons of the methylene group adjacent to the methyl terminal in the α-position –CH_2_-C**H**_2_-CH_3_ (Hb) at δ 1.34 ppm. The backbone methylene protons in the saturated chains of the fatty acid –CH_2_–C**H**_2_-CH_2_- (Hc) showed signals at δ 1.29 ppm, and the methine of the epoxy ring proton (Hd) peaks in the δ 2.77–2.99 ppm region. 

The NMR signal observed in the δ 1.44–1.47 ppm region could be attributed to the methylene groups in the α-position of the epoxy ring, and on the other side in the α position, to (EPOXY-C**H**_2_-CH_2_-) methylene groups, labeled as (He). These protons are in a different chemical environments compared with the other methylenes in the α position between two epoxy moieties (EPOXY-C**H**_2_-EPOXY) labeled as (Hf), with a resonance peak at δ 1.66–1.68 ppm, which are more deshielded due to the effect of the second epoxy group [26]. This observation reveals the presence of the epoxy linoleic fatty acid moiety in the epoxidized palm oil molecule. 

The NMR peaks at the 1.58–1.60 ppm region can be due to the resonance of the methylene protons (Hg) β to the >C=O of the glycerol backbone –C=OCH_2_-C**H**_2_, while the methylene in the α position to the carbonyl group showed a signal at δ 2.27–2.30 ppm (Hh), -C=OC**H**_2_-CH_2_. The NMR peaks at the δ 5.23–5.50 and δ 4.1–4.40 ppm region can be attributed to the methine group (Hi) and the methylene group protons (Hj, Hk) –C**H**_2_C**H**C**H**_2_- of the glycerol backbone, respectively.

**Figure 5:**
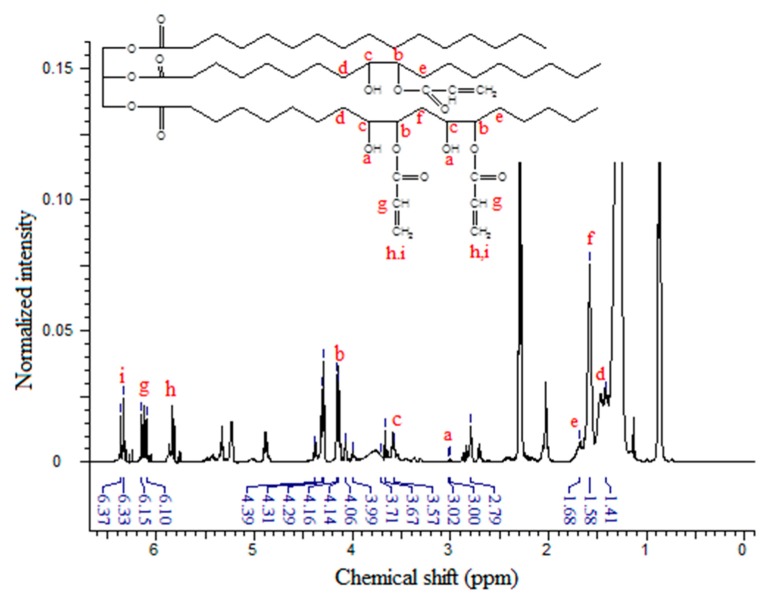
^1^H-NMR Spectrum of EPOLA.

Since the synthesis of the epoxy acrylate involves opening of the oxirane rings and grafting of the acrylate moieties on the triglyceride backbone, besides, the formation of hydroxyl groups as described above, NMR analysis can provide strong evidence by tracing the signal of these moieties and their adjacent groups. Figure 5 shows the ^1^H-NMR spectrum of EPOLA, where in addition to the characteristic signals of the triglyceride backbone, the spectrum shows the NMR signals assigned to the acrylate groups. The NMR signal with the chemical shift at δ 3.58–3.71 ppm (multiplet) can be assigned to the methine protons (Hc) in the α position of the –OH groups (-C**H**-OH), and the other methine protons (Hb) α to the oxygen atom of the acrylate groups –CH-C**H-**O-C=O<, show signals at δ 4.07–4.16 ppm (quartet). These protons (in the epoxy compound ^1^H-NMR) showed signals at δ 2.77–2.99 ppm, therefore we can state that they became more deshielded due to the stronger anisotropy effect of the adjacent oxygen groups of the hydroxyl and the ester groups, compared with the oxygen of the epoxy group. This result provides excellent evidence for the epoxy ring opening, and grafting of acrylate groups.

On the other hand, the methylene groups β to the hydroxyl and the oxygen (previously α to the epoxy rings) showed a slight difference in their chemical shift in the EPOLA spectrum *i.e.*, the Hd (OH-CH-C**H**_2_-CH-O), He (-C**H**_2_-CH-OH), and Hf (-C**H**_2_-CH-O) protons in Figure 4.

The NMR signals of the vinyl moieties associated to the acrylate groups C**H**_2_=CH- (Hh, Hi) and the methine proton (Hg) CH_2_-C**H**- were observed at δ 5.77–5.86 ppm, δ 6.33–6.37 ppm and δ 6.10–6.15 ppm, respectively. These protons are not equivalent and they have different chemical shifts due to the effect of their orientation, since (Hh) is *cis*, (Hi) is *trans* and (Hg) is *gem* [27]. Obviously, due to the opening of the epoxy rings, OH groups are formed, and thus, the NMR signals at δ 2.99–3.01 could be attributed to the resonance of the hydroxyl groups, similar to the reported observation previously [28].

Although the NMR spectrum of EPOLA showed a signal at δ 2.77 ppm related to the presence of epoxy ring residues there are still strong evidences of the successful synthesis of the epoxy acrylate. The NMR results reveal the presence of vinyl signals, which are due to the grafted acrylate groups’ absorptions and there was no contribution of free acrylic acid residues, since no carboxylic acid group protons are detected (normally at very low field of δ 10–13 ppm).

The acrylation percentage and number of acrylate groups per triglyceride molecule were calculated from the integrated peak areas taking the methyl signals as an internal reference standard. Equation (2) was used in the calculation of the acrylation percentage; similar to the method described previously in [29]:

(2) $$\text{Acrylation \%}=\frac{{\text{A}}_{\text{Epoxy}}}{{\text{A}}_{\text{Epoxy }}+{\text{ A}}_{\text{Acrylate}}}\;\times \;100$$

where, A_Epoxy_ is the integrated area of the methine epoxy protons at δ 2.77 ppm, and the A_Acrylate_ is the integrated peak area of the acrylate protons at δ 5.77–6.37 ppm; the acrylation percent was found to be 82.07%. The EPOP and EPOLA NMR results were found to be in a good agreement with the results obtained from FTIR and quite similar to the previously reported NMR results of epoxy oil acrylates [30,31,32].

**Figure 6:**
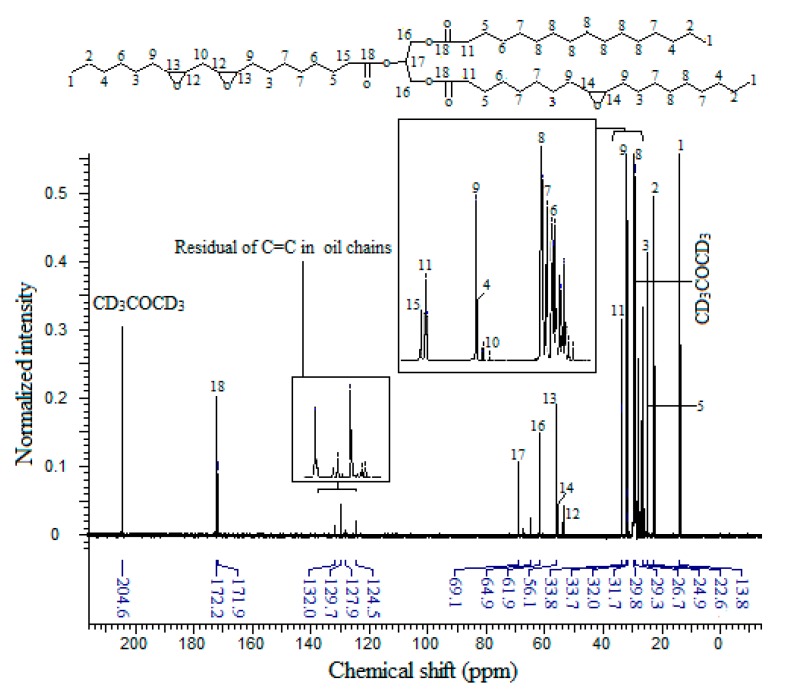
^13^C-NMR Spectrum of EPOP.

The ^13^C-NMR spectrum of EPOP is displayed by Figure 6, where the acetone-d_6_ carbons show two signals, the first one at δ 29.0 ppm corresponding to the methyl groups and the signal of the carbonyl group appears at δ 204.6 ppm. 

The NMR peak at δ 13.8 ppm is related to the carbon atom of the methyl group at the terminus of the fatty acid backbone, –CH_2_-**C**H_3_ (C1), while the methylene groups showed different chemical shifts and peak positions due to the effects of the adjacent groups, and their position with respect to each functional group (δ, β, γ….). The carbon of the methylene groups in the position α to the methyl terminal groups (C2) –**C**H_2_-CH_3_ have an NMR peak at δ 22.6 ppm, while the methylene carbons β to the methyl (C4) absorbed at δ 31.7 ppm. The methylene in the γ position with respect to the CH_3_ groups (C7) appear at δ 29.7 ppm and methylene carbon (C6) shows a signal at δ 29.6 ppm.

The carbon of the methylene (C6) in the position γ to the carboxyl groups of the glycerol part >C=OCH_2_CH_2_**C**H_2_- showed a signal at δ 29.6 ppm, while the β carbon (>C=OCH_2_**C**H_2_CH_2_-) (C5) showed a peak at δ 24.9 ppm. The other methylene groups of the saturated fatty acid backbone (C8) appear at δ 29.8 ppm.

The carbon atoms of the epoxy rings (C13, C14 and C12) were found to have NMR signals in the δ 53.7–56.1 ppm region [33,34], while the carbon atom of the methylene group between the two epoxy moieties in the linoleic chain (C10) shows a resonance at δ 31.5 ppm. On the other hand, the methylene groups at the α position with respect to the epoxy groups (epoxy-**C**H_2_-C9) have NMR signals at δ 32.0 ppm; these groups are shifted downfield due to the electronegativity of the adjacent group. Similarly, the methylenes in the α position of the carboxylic acid groups of the glycerol part, C11 and C15, have signals at δ 33.7 and 33.8 ppm, respectively. The glycerol backbone contains two methylene groups (C16) with signals at δ 61.9 ppm, one methine group (C17) has a signal at δ 69.1 ppm, and the carbons of the carboxylic acid groups (C18) appear in the δ 171.9–172.2 ppm region.

According to the overall analysis of the ^13^C-NMR spectrum of EPOP, results were consistent with the proton NMR and FTIR results. However, the spectrum showed some signals related to the presence of unsaturated carbon (sp_2_) of the palm oil fatty acids *i.e.*, a signal in the δ 129.6 ppm region for (>C=C<) moieties, and at δ 27.0–28.0 ppm signal assigned to α methylene groups attached to the unsaturated carbon atoms (>C=C-**C**H_2_-).

**Figure 7:**
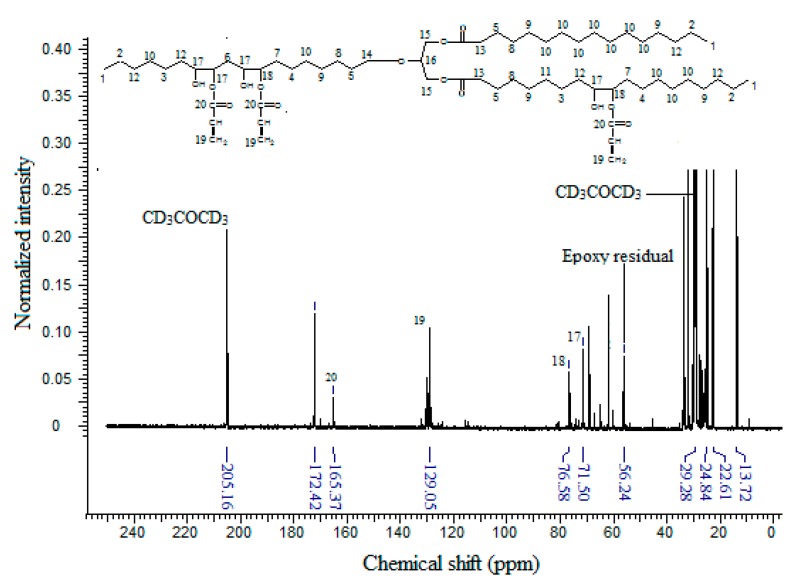
^13^C-NMR Spectrum of EPOLA.

The ^13^C-NMR spectrum of EPOLA (Figure 7) showed that the carbon atoms of the acrylate moieties (C19) have NMR signals in the δ 129.0–129.9 ppm region, while the carbonyl carbon atoms of the acrylate groups (C20), showed a resonance signal at δ 166.3 ppm. In addition to the presence of the acrylate carbons, tracing the carbon atoms C17 and C18, which are in the α position of the hydroxyl group and the ester groups, respectively, these show resonances in the δ 71.5–77.5 ppm region. These carbons originate from the oxirane rings, and they previously showed NMR signals in the δ 53 to 66 ppm region (Figure 6), therefore they became more deshielded due to the stronger anisotropy of the new adjacent hydroxyl and ester groups. These results indicate clearly that the grafting of the acrylate groups on the backbone was achieved successfully.

### 2.3. Characterization of the Solid Cured Films of EPOLA

Mixtures of EPOLA resins, reactive acrylate monomers and 4% photoinitiator were exposed to UV radiation for different exposure times to obtain tack-free solid films. Free radical polymerization of EPOLA took place rapidly upon the exposure to UV radiation leading to formation of cross-linked networks; a UV radiation dose about 0.351 mJ/cm^2^ was found to be sufficient to obtain solid films.

**Figure 8:**
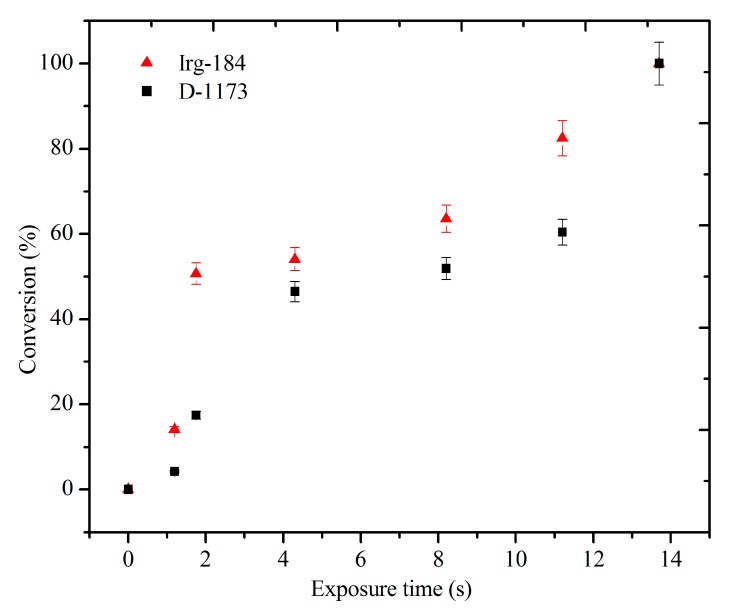
The Conversion of C=C in EPOLA UV cured Films (**■**) Photoinitiator D-1173 (**▲**) Photoinitiator Irg-184.

The curing behavior at different exposure times was studied by tracing the intensity of the FTIR band at 810 cm^−1^ corresponding to the acrylate vinyl groups. The peak intensity decreased with increasing exposure time to UV radiation, indicating the consumption of the C=C double bonds in the polymerization reaction. The correlation between the exposure time and the percent conversion of the vinyl C=C using two types of photoinitiators, *i.e.*, D-1173 and Irg-184, is shown in Figure 8.

It was observed that when the EPOLA films containing D-1173 as a photoinitiator were exposed to UV radiation for about 11.2 s, the percent conversion of the vinyl groups was about 60.3%, while, using Irg-184 for a similar exposure time led to a higher conversion of about 82.4%. These results imply that using Irg-184 to initiate the free radical polymerization of EPOLA, increases the chain transfer and the termination rate [35], which lead to higher conversion, compared to D-1173, therefore it is more suitable for obtaining better curing of the EPOLA resin. However, extending the exposure time to 13.7 s led to the disappearance of the FTIR band at 810 cm^−1^, indicating the total consumption of the vinyl groups, and the conversion percentage was 100% for both EPOLA films.

**Figure 9:**
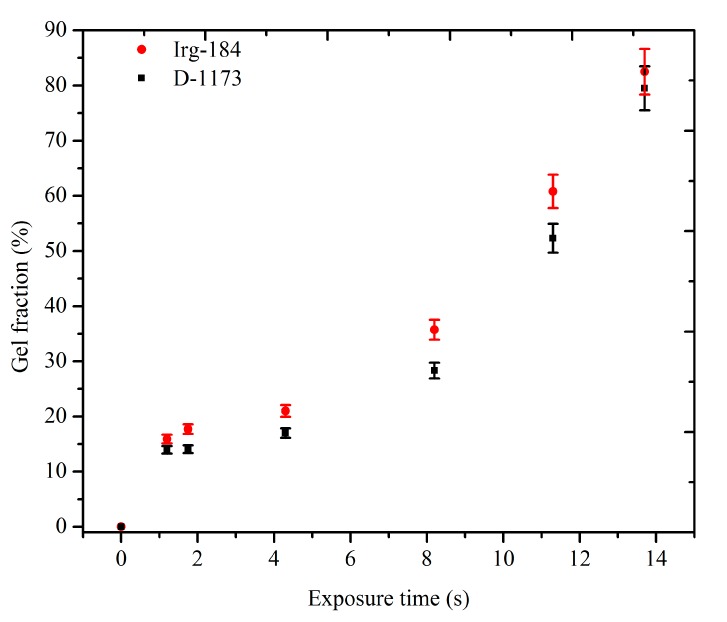
Gel Fraction Percentages of the UV cured EPOLA Films (**■**) Photoinitiator D-1173 (**●**) Photoinitiator Irg-184.

Figure 9 shows the gel fractions of the cured films against the exposure time to UV radiation. It was observed that the cured films obtained using Irg-184 as a photoinitiator, showed higher gel fraction percentages than those containing D-1173. This observation indicates the higher cross-linking density of the first films, and is in good agreement with the results of the conversion. The cross-linking densities were increased as the exposure time increased, which could be attributed to the increase of the deposited energy in the matrix, and resulted in a higher free radicals concentration. The highest cross-linking densities were obtained after 13.7 s exposure time, were about 79.5% and 82.5% of the D-1173 and Irg-184 films, respectively.

**Figure 10:**
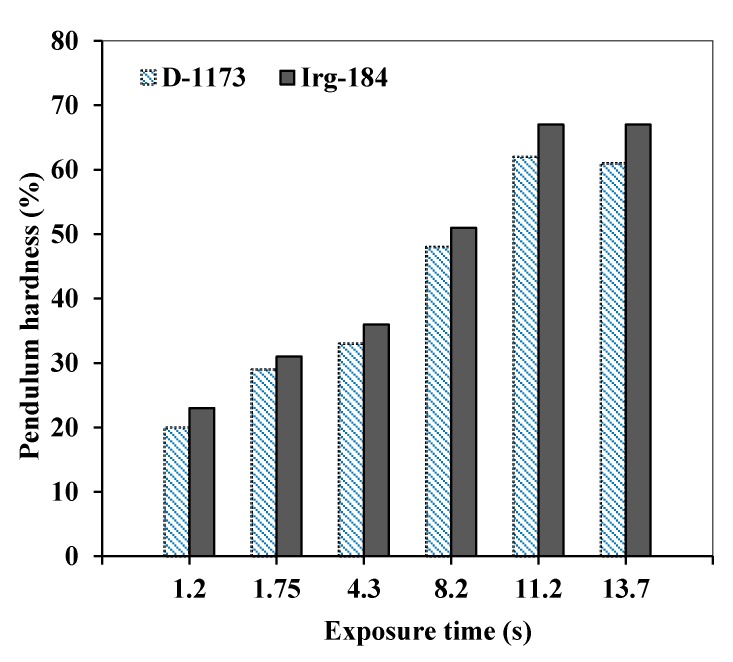
Pendulum Hardness Percentages of the UV cured EPOLA Films with D-1173 and Irg-184 Photoinitiators.

Similarly, the hardness of the cured EPOLA films shows better results when Irg-184 was used as photoinitiator (Figure 10), which is consistent with the higher conversion rate and cross-linking density, compared to the results obtained by using D-1173 photoinitiator. The hardness percentage was higher than 60% when EPOLA films exposed to UV radiation for 11.2 s, and was found to better than EPOLA hardness results reported previously [24]. Therefore, the overall results revealed that EPOLA films showed better performance when Irg-184 was used as photoinitiator, and it is more suitable than D-1173 to obtain better curing results for potential uses in coating applications.

**Figure 11:**
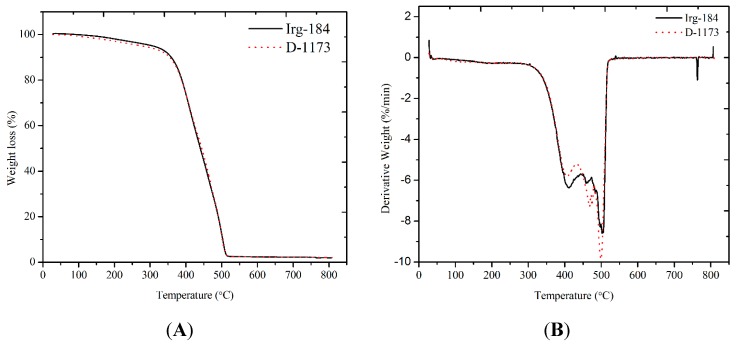
(**A**) TGA Thermograms and (**B**) Derivatives Curves of EPOLA Films with D-1173 and Irg-184 Photoinitiators.

The thermal properties of EPOLA films were characterized by thermogravimetric analysis (TGA) under a N_2_ atmosphere. Figure 11A shows the TGA traces of EPOLA films, the weight derivatives curves (DTG) are shown in Figure 11B and data are collected in Table 4.

**Figure 12:**
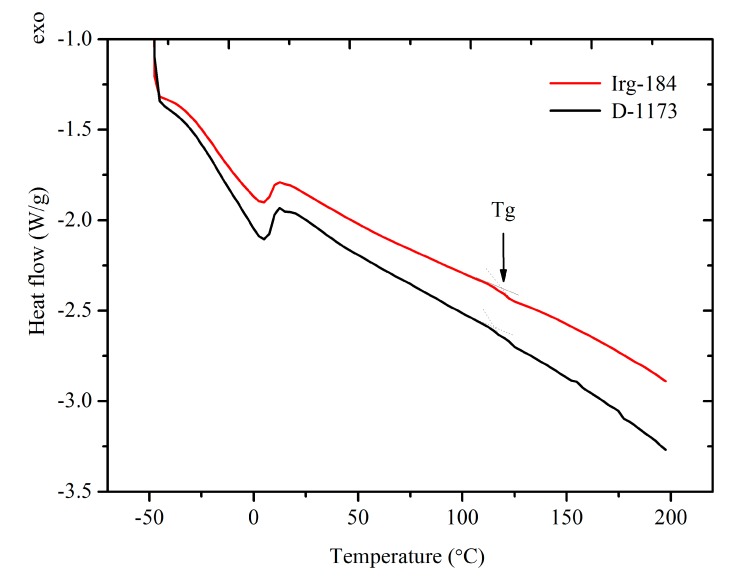
DSC Therograms of EPOLA Films Containing Irg-184 and D-1173 photoinitiators.

It has been observed that EPOLA films showed significant difference in the T_5__%_ (temperature of 5% weight loss) values, and Irg-184 film showed a higher value by 31 °C, compared to D-1173 film. This result implies that the initial degradation temperature of the first film was higher than the second one, indicating a better thermal stability, which can be due to the higher cross-linking density. Moreover, the T_max_ (temperature of maximum degradation rate) showed higher value, confirming the higher thermal stability of the first films. In general, both EPOLA films showed similar degradation behavior, and lost a very small percentage of their weight in the temperature range below 200 °C, which can be attributed to the decomposition of the volatile components (*i.e.*, unreacted monomers and photoinitiator). The main degradation step was observed in the temperature range between 250 °C and 550 °C, related to the decomposition of the unreacted palm oil epoxy fatty acids, at a lower temperature compared to the polymerized epoxy acrylate parts, which are expected to dissociate at higher temperature [36]. In addition, the char yields at 600 °C were found to be around 2.3% for both EPOLA films. Figure 12 shows the DSC thermograms of EPOLA films containing Irg-184 and D-1173 photoinitiators. It was observed that both films showed only one glass transition temperature (*Tg*), indicating that both systems have uniform structures. The *Tg* value (Table 4) of EPOLA film containing Irg-184 was about 119.6 °C, slightly higher than the value of EPOLA containing D-1173 (115.5 °C). This can be attributed to the higher cross-linking density in the first film, which leads to reduce the chain motion. These values are quiet similar to those reported by Kardar *et al.* [37].

**Table 4:** Thermal data of EPOLA Films Containing Irg-184 and D-1173 Photoinitiators.

EPOLA	T_5%_/°C	T_10%_/°C	T_50%_/°C	T_max_/°C	*Tg*/°C	Char (%)
Irg-184	308.5	359.2	440.3	506.0	119.6	2.34
D-1173	277.0	354.0	444.0	496.0	115.5	2.30

In summary, the obtained results showed that the thermal stability is better than that of the previously prepared UV-curable epoxy acrylates including arylene ether sulfone linkages. However, they reported higher solid residue percentages at 600 °C, revealing that the additional functional groups significantly improved the thermal and thermo-oxidative properties at high temperature [38]. Similar findings were also reported in curable systems including phthalonitrile-functional phthalazazine, which indicated also the potential improvement of the thermal and thermo-oxidative stability by introducing such functional groups in the curable systems [39].

**Figure 13:**
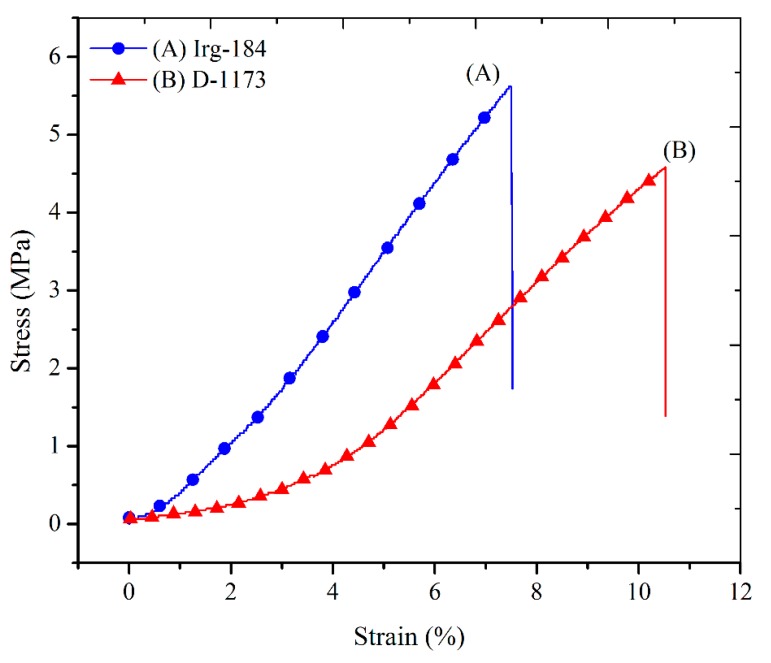
Stress-strain Curves of EPOLA Films Containing Irg-184 and D-1173 Photoinitiators.

The stress-strain curves of EPOLA films are shown in Figure 13. The typical values of the tensile strength, Young’s modulus and the elongation at break percentages are presented in Table 5. It could be observed that EPOLA film containing Irg-184 has a higher tensile strength value compared to the film with D-1173. This result is compatible with the higher cross-linking density of the first films, which is attributed to the strong tethering between the epoxy acrylate chains, and therefore, higher stiffness. The Young’s modulus values showed more pronounced differences between the two EPOLA films, while the elongation at break was lower in the first film, confirming the higher stiffness due to the strong cross-linking. The obtained tensile values were found to be higher than those of a previously reported vernonia oil-based epoxy acrylate [10], while, Li *et al.*, reported better mechanical properties when they investigated a soybean oil-based epoxy acrylate [40].

**Table 5:** Tensile strength, Young’s Modulus and Elongation at break of EPOLA Films Containing Irg-184 and D-1173 Photoinitiators.

EPOLA	Tensile Strength (MPa)	Young’s Modulus (MPa)	Elongation at Break (%)
Irg-184	6.2(±0.7)	65.7(±5.0)	8.7(±0.15)
D-1173	5.2(±1.2)	53.6(±2.3)	12.5(±0.19)

## 3. Experimental Section 

### 3.1. Materials 

Epoxidized palm oil product (EPOP) with oxirane-oxygen content = 3% per gram, iodine value = 0.76 I_2_/100 g was purchased from Intermed Sdn. Bhd. (Kuala Lumpur, Malaysia). Acrylic acid (99%, Sigma-Aldrich, St. Louis, MO, USA). Triethylamine (TEA) and 4-ethoxyphenol (4-MP) were supplied by Aldrich Chemical Co. Inc. (Milwaukee, WI, USA). 1,1,1-Trimethylolpropane triacrylate (TMPTA), dipropylene glycol diacrylate (DPGDA), were purchased from Cytec Industry (Vlaardingen, The Netherlands). The photoinitiators 1-hydroxycyclohexyl phenyl ketone (Irgacure^®^ 184) and 2-hydroxy-2-methyl-1-phenylpropan-1-one Darocur^®^ 1173 (D-1173), were supplied by Ciba-Geigy Specialty Chemicals (Basel, Switzerland). Acetone (analytical grade) was purchased from Merck (Darmstadt, Germany).

### 3.2. Synthesis of Epoxidized Palm Oil Acrylate

EPOP (0.79 mol, 1000 g) was measured and placed in a four necked flask equipped with a mechanical stirrer, condenser and thermometer. About 1% (of the EPOLA weight) of both 4-methoxyphenol (an inhibitor to prevent premature polymerization of the reactants) and triethylamine (TEA) as a catalyst were added to the reaction flask and was stirred at 40 °C for 1 h. Under continuous stirring, acrylic acid (1.2 moles, 90 g) was added to the reactor dropwise (1 drop/s) to control the reaction progress and avoid homo-polymerization of the acrylic acid and the temperature of the reaction was gradually raised to 110 °C. The acrylation reaction was followed by measuring the acid value and following the functional groups’ IR absorptions using Fourier Transform Infrared Spectroscopy (FTIR). EPOLA was obtained after 16 h when the acid value of the reaction mixture was less than 15 mg KOH/g (~10.2 mg KOH/g). EPOLA was purified by washing with distilled water and equilibrated overnight to remove unreacted acrylic acid, inhibitor and catalyst residuals. 

### 3.3. Preparation of EPOlA Cured Films by UV Free Radical Polymerization 

Solid EPOLA films were obtained via free radical polymerization of the vinyl C=C of the acrylate groups, using the ultraviolet (UV) radiation curing technique. The coating formulae were comprised of EPOLA (7 g), TMPTA (10 phr), DPGDA (30 phr) and the photoinitiators D-1173 or Irg-184 (4% of the total formula weight). Wet formulae were coated in glass substrates with a thickness around 12 ± 2 µm using a wired bar hand applicator, then they were exposed to UV radiation for different exposure times using a UV curing machine equipped with a mercury lamp with intensity about 80 Watt/cm (manufactured by IST Stralentechnik, Nuertingen, Germany). The conveyor speed of the UV machine was set to be 5 m/min and the current was 7.5 A).

### 3.4. Characterization of Epoxidized Palm Oil Acrylate

The acid value was determined by direct titration of the resin against KOH 0.1 N, with 5 drops of the indicator bromothymol blue: phenol red (1:1 in 90% ethanol solution), and was calculated using the following equation:

(3) $$\text{AV}=\;\frac{5.61\;\times \text{ f }\times \text{ v}}{\text{w}}$$

where, f = titration factor of the KOH, (f = 1), V = volume of the 0.1 N KOH solution required for titration the acid and W = the weight of the resin sample.

Oxirane-oxygen (Ox-O_2_) values were measured following the AOCS-CD-63 standard method. Samples were tiaagainst 0.1 N HBr using crystal violet indicator (5 drops). The results were obtained as a percentage of oxirane ring per gram of resin as follows:

(4) $$\text{Ox}-{\text{O}}_{2}\text{\%}=\;\frac{1.6\;\times \text{ N }\times \text{ v}}{\text{w}}$$

where N = the normality of HBr, V = the volume of HBr required for titration. W = weight of the sample. 

Hydroxyl value (OH value) was determined by following the ASTM D4274 standard. The OH values were calculated using the following Equation (5) and results were reported as mg KOH/g resin:

(5) $$\text{OH No}.\;=\frac{\left[\left(\text{A}-\text{B}\right)\text{ N }\times 56.1\right]}{\text{W}}$$

where, A = NaOH required for titration of the resin sample (mL). B = NaOH required for titration of the blank (mL). N = normality of the NaOH solution, and W = the weight of resin sample. 

The Iodine value was determined according to the official method AOCS CD 1D-92 (Wijs solution method) and calculated as:

(6) $$\text{Iodine value}=\;\frac{\left(\text{B}-\text{S}\right)\;\times \text{ N }\times \;12.69}{\text{W}}$$

where, B = sodium thiosulfate required for titration of the black (mL). S = sodium thiosulfate required for titration of the sample. N = the normality of sodium thiosulfate solution, and W = the weight (g) of the resin sample.

The viscosity of the resins was determined by using a model RVTDV-IICP viscometer (Brookfield, Middleboro, MA, USA) at 25 °C. The density was determined according to the standard test method ASTM D1475-98 (reapproved in 2012). Molecular weights and molecular weight distributions of EPOP and EPOLA were determined by a Gel Permeation Chromatography (GPC) system (PolyLab LC-chromatograph, Houston, TX, USA), equipped with refractive index, light scattering and viscometer detectors.

The chemical structure of the resins was investigated by FTIR spectrophotometry (Spectrum 2000, Perkin ElmerWaltham, MA, USA), using KBr disks and the attenuated total refraction (ATR) method. FTIR spectra were recorded in the wavenumber range between 400 to 4000 cm^−1^ with a total of four scans.

^1^H-NMR and ^13^C-NMR measurements were conducted on a JNM-ECX 500 NMR spectrometer (JEOL, Peabody, MA, USA). Samples were dissolved in deuterated acetone (acetone-d_6_). Eight scans were performed to measure the samples in a magnetic field of 11.74 T and field strength of 500 MHz.

The cross-linking density of the solid films was obtained by measuring the gel fractions. Samples accurately weighed in a wire mesh extracted by Soxhlet using acetone at 70 °C for 24 h. Extracted samples were dried in a vacuum oven for 8 h at 80 °C and then in a normal oven at 80 °C for two days, until consistent weights were obtained. Gel fraction percentages were calculated as:

(7) $$\text{Gel fraction \% }=\;\frac{\text{B}}{\text{A}}\;\times \;100$$

where, A and B are sample weight before and after extraction, respectively.

The curing behavior was investigated by following the IR absorption band at 810 cm^−1^ in the FTIR spectrum, corresponding to acrylate C=C twisting, of the liquid and cured films, at different exposure times to the UV radiation. The conversion percentages were calculated using Equation (8):

(8) $$\text{Conversion\% }=\;\frac{{\text{A}}_{0}-{\text{A}}_{\text{t}}}{{\text{A}}_{0}}\;\times \;100$$

where, ${\text{A}}_{0}\;$ is the C=C absorption peak intensity at 810 cm−1 before exposure to UV energy (uncured film), and ${\text{A}}_{\text{t}}$ is the C=C peak intensity after an exposure time (t) (the CH_3_ peak at 2,924 cm^−1^ was used as an internal standard) [41]. 

UV-cured film hardness was measured by the Pendulum hardness tester; model BYK-Labotron, (Wesel, Germany), according to the standard method ASTM D-4366-87, pendulum damping (König test method). Results were expressed by percentage of pendulum hardness (PH%) to the glass standard, and obtained by the following equation:

(9) $$\text{P}.\text{H \%}=\frac{\text{Sample }\left(\text{osc}\right)}{\text{ Standard glass }\left(\text{osc}\right)}\;\times \;100$$

where, Sample (osc) is the number of oscillations for damping from 6° to 3° of the sample. Standard glass (osc) is the number of the oscillations of the standard glass. 

The solid EPOLA films’ thermal properties were measured by thermogravimetic analysis (TGA) using a TGA-SDTA 851e instrument (Mettler Toledo, Columbus, OH, USA). Samples were analyzed in temperature range from room temperature to 800 °C, at heating rate of 10 °C/min, under a N_2_ atmosphere. Differential scanning calorimetry (DSC) was carried out using a DSC 822 (Mettler Toledo). Samples were scanned at 5 °C/min, in the range from −50 °C to 200 °C.

The mechanical properties of EPOLA films *i.e.*, tensile strength, Young’s modulus and elongation at break percentage, were measured using a tensile machine (Strograph E3-Toyo Seiski, Tokyo, Japan). Samples were analyzed according to the ASTM 1822L standard. Specimens were cut in a dumbbell shape according to the dimensions determined by the standard. The cross head speed was 5 m/min, the overhead load was 0.5 kN. 

## 4. Conclusions

In this article, the acrylation of the epoxidized palm oil to produce EPOLA a yield of about 82% was successfully accomplished. Spectroscopic investigations by means of FTIR and NMR revealed that the oxirane rings were opened, and the acrylate groups were grafted onto the triglyceride molecules. Accordingly, the molecular weight of EPOP was increased as well as its viscosity. UV radiation curing of EPOLA was also investigated, using two types of photoinitiators, D-1173 and Irg-184. EPOLA film with Irg-184 showed higher conversion rates, cross-linking density and hardness percentages. Moreover, the mechanical and the thermal properties of this film showed superior results compared to EPOLA film made with D-1173. Therefore, Irg-184 was found to be more suitable, and resulted in better curing results.
